# Cost-Effectiveness of Dengue Vaccination Programs in Brazil

**DOI:** 10.4269/ajtmh.16-0810

**Published:** 2017-05-03

**Authors:** Eunha Shim

**Affiliations:** 1 Department of Mathematics, Soongsil University, Seoul, Republic of Korea

## Abstract

The first approved dengue vaccine, CYD-TDV, a chimeric, live-attenuated, tetravalent dengue virus vaccine, was recently licensed in 13 countries, including Brazil. In light of recent vaccine approval, we modeled the cost-effectiveness of potential vaccination policies mathematically based on data from recent vaccine efficacy trials that indicated that vaccine efficacy was lower in seronegative individuals than in seropositive individuals. In our analysis, we investigated several vaccination programs, including routine vaccination, with various vaccine coverage levels and those with and without large catch-up campaigns. As it is unclear whether the vaccine protects against infection or just against disease, our model incorporated both direct and indirect effects of vaccination. We found that in the presence of vaccine-induced indirect protection, the cost-effectiveness of dengue vaccination decreased with increasing vaccine coverage levels because the marginal returns of herd immunity decreases with vaccine coverage. All routine dengue vaccination programs that we considered were cost-effective, reducing dengue incidence significantly. Specifically, a routine dengue vaccination of 9-year-olds would be cost-effective when the cost of vaccination per individual is less than $262. Furthermore, the combination of routine vaccination and large catch-up campaigns resulted in a greater reduction of dengue burden (by up to 93%) than routine vaccination alone, making it a cost-effective intervention as long as the cost per course of vaccination is $255 or less. Our results show that dengue vaccination would be cost-effective in Brazil even with a relatively low vaccine efficacy in seronegative individuals.

## Introduction

Dengue is a febrile illness caused by any one of the four serotypes of dengue virus (DENV-1, DENV-2, DENV-3, or DENV-4).^1^ The disease is transmitted from human to human through the bite of mosquitoes of the genus *Aedes*.^2^ Dengue is endemic in more than 100 countries, and nearly 4 billion people are at risk for dengue, with 390 million dengue infections occurring every year.^3^

The outcomes of dengue infection range from asymptomatic and subclinical to symptomatic infections.^4^ Symptomatic infections vary from a mild, flu-like illness known as dengue fever (DF) to severe dengue, such as dengue hemorrhagic fever (DHF) and dengue shock syndrome (DSS).^2^ Infection with dengue virus provides long-term protection against the particular serotype that caused the disease. However, dengue infection by one serotype provides only short-lived immunity to the other three dengue virus serotypes.^5^ Furthermore, individuals experiencing their second natural dengue infection have a higher risk of severe disease than those experiencing primary infections, an effect referred to as antibody-dependent enhancement (ADE).^6^

Although six vaccines are in clinical development, to date, only the Sanofi–Pasteur vaccine, Dengvaxia, has completed phase III trials and has been licensed in 13 countries—Mexico, the Philippines, Brazil, El Salvador, Costa Rica, Paraguay, Guatemala, Peru, Indonesia, Thailand, Singapore, Bolivia, and Cambodia.^7^,^8^ Dengvaxia is a chimeric, live-attenuated, tetravalent dengue virus vaccine (CYD-TDV), based on the licensed yellow fever vaccine, 17D.^9^ A large phase III randomized, controlled vaccine trial of CYD-TDV in Latin America was reported in 2014.^10^,^11^ The overall efficacy of the vaccine against virologically confirmed dengue cases was 64.7% (95% confidence interval [CI] = 58.7, 69.8); however, the efficacy varies depending on serostatus of an individual. Specifically, vaccine efficacy for confirmed cases of dengue was lower in seronegative individuals (52.5%; 95% CI = 5.9, 76.1) with *N* = 387 for vaccine group and *N* = 208 for control group) than in seropositive individuals (81.9%; 95% CI = 67.2, 90.0) with *N* = 1,560 for vaccine group and *N* = 763 for control group.^11^–^13^ Furthermore, analysis of the phase III trials of Dengvaxia suggests that there would be an increased risk of hospital admissions that would accompany breakthrough dengue infections in vaccinated seronegative individuals despite the vaccine providing high rates of protection in vaccinated partially dengue-immune individuals (i.e., seropositive).^14^

In light of the recent approval of the administration of CYD-TDV and its variable efficacy, it is essential to consider the cost-effectiveness of dengue vaccination in Brazil. To date, just a few studies on the cost-effectiveness of a hypothetical dengue vaccine have been published.^15^–^21^ Only one study evaluated the cost-effectiveness of dengue vaccination in Brazil,^18^ whereas another study evaluated the economic burden of dengue in Brazil.^22^ Prior studies predicted that dengue vaccination would be cost-effective up to a total vaccination cost of $200 and $237 in Thailand and Brazil, respectively.^16^,^18^ In the Philippines, dengue vaccination was shown to be cost-effective at costs up to $72, whereas in Singapore, dengue vaccines would be cost-effective under $53 assuming 10-year vaccine-induced immunity.^17^,^21^ Although these studies provide valuable evidence that the vaccine would be cost-effective,^15^–^21^ a substantial amount of additional information has emerged recently, including vaccine safety and efficacy, as well as the target ages of vaccination. This new information has not been addressed in prior cost-effectiveness analyses of dengue vaccination in Brazil.^18^

Herein, we evaluated the cost-effectiveness of dengue vaccination in Brazil, taking into account the reduced vaccine efficacy in seronegative recipients and various vaccination strategies. For this purpose, we used an age-structured model of dengue transmission and vaccination^23^ and fit it to the data on dengue incidence to examine the potential cost-effectiveness of deploying a dengue vaccine in Brazil. We first estimated the economic and epidemiological impact of dengue vaccination and then calculated its cost-effectiveness at various vaccine costs with and without a catch-up vaccination program. Furthermore, we identified a threshold vaccine cost at which the dengue vaccine becomes cost-effective.

## Methods

### Overview.

We considered vaccine interventions using a modified age-structured model of dengue transmission^23^ (Table 1, Supplemental Appendix). Our model reflects the current understanding of the natural history of dengue, and it is a model of the vaccine based on the general results for Dengvaxia. Our model considers the direct effects of vaccination as well as the indirect protective effect of herd immunity for individuals who are not vaccinated.

Dengue infection produces lifelong immunity to the infecting serotype and induces temporary cross-protection against other serotypes, but dengue infection can later enhance disease severity in subsequent infections, due to an effect of ADE. We represent multiple primary, secondary, and tertiary infections with different disease outcome probabilities depending on the number of previous infections. It is known that two prior dengue infections provide protective immunity against severe dengue disease in a subsequent infection.^24^,^25^ Therefore, our model assumes that third infections from dengue are asymptomatic.^24^,^26^–^28^ We classify infections as asymptomatic (“unapparent”) or symptomatic (a “case”). The proportion, *g*_*x*_, of infected individuals is assumed to be symptomatic, where the subscript *x* refers to the epidemiological status of individuals. Symptomatic infections are further separated into mild or severe cases. We assume that the probability of severe disease is dependent on infection history and vaccination status, consistent with empirical data.^18^,^28^,^29^ Specifically, we account for ADE by assuming that the probability of severe disease after a secondary infection is greater than after a primary infection.^29^,^30^ Therefore, our model considers both cross-protection (i.e., no risk of developing a heterotypic infection for a limited time after an infection) and cross-enhancement (i.e., differential risk of developing severe cases on primary, secondary, and tertiary infection). The dengue infection model and the calculation of economic burden associated with dengue infection and vaccination are described in Supplemental Appendix.

### Epidemiological parameters.

The force of infection is

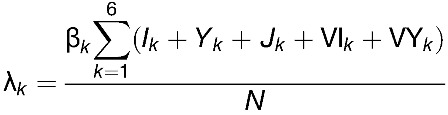
where β_*k*_ is the age-dependent transmission rate among age group *k* (Supplemental Figure 1). We also assumed symptomatic and asymptomatic individuals are equally infectious. Our model combines the underlying process of infection in the vector and subsequent transmission to other humans into one aggregate rate, β_*k*_.29

Cases of dengue in Brazil are known to be substantially underreported.^31^ To fully account for cases of dengue, we ran the model using baseline parameters to equilibrium and calibrated our model to an adjusted annual dengue incidence rate of 2.03% (including both symptomatic and asymptomatic infections), an annual incidence of DF of 1.07%, and an annual incidence of DHF/DSS of 0.029%, which incorporates underreported cases^18^,^32^,^33^. The transmission rates (β_*k*_) were chosen to capture the patterns of empirical dengue incidence in Brazil. Specifically, such age-specific incidence profiles (Figure 2) were obtained using β_k_ values (Table 2). Therefore, our initial conditions were determined based on the endemic equilibrium of our model. Infected individuals are assumed to recover from primary infections at rate γ and gain clinical cross-protection, which prevents clinical illness but allows seroconversion. The average duration of clinical cross-protection is assumed to be 1/γ_*C*_ (Table 2).

### Vaccine-related parameters.

In our model, vaccination reduces the probability of infection with dengue-given exposure, and has no other direct effect on transmission. We assumed that the vaccine efficacy was consistent with the phase III trial results for CYD-TDV in Latin America^10^,^11^ (Table 2). Trial results indicated that an individual's serostatus before vaccination affects vaccine efficacy.^41^ Specifically, prior dengue infection was shown to increase vaccine efficacy.^41^,^42^ Such effects were incorporated into our model (ε < δ), where we define ε as vaccine efficacy among individuals aged nine and over who had never been exposed to dengue (referred to as seronegative individuals), whereas δ denotes the vaccine efficacy among individuals aged nine and over who had previously been exposed dengue virus (referred to as seropositive individuals).

Furthermore, our model incorporates not only vaccine-induced protection but also vaccine-enhanced dengue disease among vaccine recipients, as observed in the CYD-TDV trials.^10^,^24^ Results of phase III efficacy trials of CYD-TDV conducted in Latin America demonstrated that vaccination may present immunological similarities to an attenuated subclinical primary infection; thus, vaccination of seronegative individuals potentially increases the risk of DHF during a subsequent wild-type infection.^41^ Therefore, in our model, the probability of developing DHF/DSS after primary symptomatic infection among unvaccinated individuals was assumed to be lower than individuals who were seronegative when vaccinated (*q*_*I*_ < *q*_VI_). Here, *q*_*I*_ and *q*_VI_ are defined as the probability of developing DHF among symptomatically infected individuals in *I*_*k*_ and VI_*k*_, respectively (Table 2).

### Vaccination policies considered.

In prior studies, many national vaccination strategies were tested: routine vaccination at various ages, including 9-year-olds, considering reports of pooled vaccine efficacy for 9–16 years of age,^12^ and catch-up immunization campaigns for various age groups.^13^,^34^ In our paper, the default policy was routine vaccination of 9-year-old children, the youngest age within the approved age range (9–45 years) for the Dengvaxia, with 70% vaccine coverage level. For sensitivity analysis, we examined the impact of lower (50%) and higher (90%) vaccine coverage levels with routine vaccination in terms of cost-effectiveness. Alternative strategies examined were 1 year of catch-up campaigns for various age groups followed by routine vaccination of 9-year-olds. For catch-up campaigns, we considered ages 10–18, 10–25, 10–35, and 10–45 as the targeted ages. For all catch-up campaigns considered, it was assumed that 70% of 9-year-olds received routine vaccination and 50% vaccine coverage was achieved in the catch-up campaigns.

### Costs.

To calculate costs, we assumed that a fraction of DF patients seek medical care, requiring ambulatory care (Table 3). For DHF cases, we assumed that hospitalization was required. The probability of developing DHF/DSS after symptomatic infections is assumed to be dependent on the serostatus and the vaccination status of each individual.

### Economic and health outcomes.

We identified many epidemiological and economic parameters from the literature and publicly available data (Tables 2 and 4). Using those parameters, we evaluated the epidemiological impact of vaccination strategies by contrasting the projected dengue burden with and without vaccine deployment over a 10-year forecasted period. To calculate health outcomes, we calculated the time-discounted disability-adjusted life-years (DALYs) lost to DF, DHF/DSS, and dengue-related deaths. Our estimated health impact is presented in quality-adjusted life-years (QALYs), by assuming that one DALY averted is equivalent to one QALY gained, as in previous studies.^49^–^51^

We calculated the total costs accrued due to vaccination and medical treatment. These monetary costs were calculated in 2017 U.S. dollars based on the Consumer Price Index for Medical Costs.^52^ To calculate the incremental cost-effectiveness ratios from a health-care perspective, we included direct medical costs and health outcomes and used an annual discount rate of 3% over a 10-year period.^53^

### Cost-effectiveness of vaccination policies.

To determine the net QALYs gained, we subtracted the total QALYs lost across the population under the vaccination scenario from the total QALYs lost across the population under the no-vaccination scenario (see Supplemental Appendix). To determine the net costs accrued, we subtracted the total costs accrued under the no-vaccination scenario from the total costs accrued under the vaccination scenario. Because both vaccine procurement and delivery costs were unknown, we varied the vaccination cost, including the cost of vaccine doses, the costs of vaccine delivery and administration, and the cost associated with vaccine wastage.

The Brazilian guidelines for health technology assessments do not specify a threshold to determine whether an intervention is cost-effective, and thus we use the threshold derived from the World Health Organization Commission on Macroeconomics and Health.^54^ In addition, we assumed that society's willingness to pay (WTP) for one DALY averted is equivalent to its WTP for one QALY, as in previous studies.^49^–^51^ Therefore, in our analysis, we assumed that interventions that would gain one additional QALY for less than three times the average per capita gross domestic product (GDP), $25,620, were deemed “cost-effective,” and those that would gain one additional QALY for less than the average per capita GDP ($8,540) were deemed “very cost-effective”^54^,^55^. The costs and utility weights used in the analysis are presented in Table 4.

## Results

**Figure 1.**
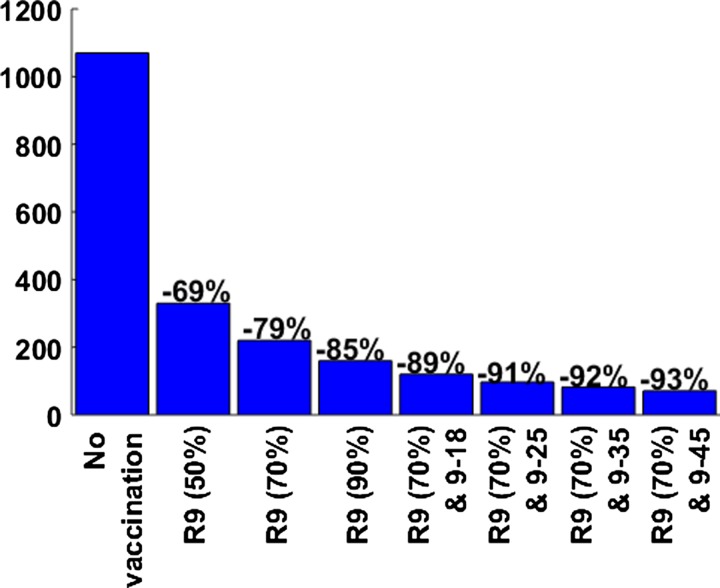
Expected yearly incidence of symptomatic cases of dengue per 100,000 for the different vaccination strategies. Incidence was averaged over a 10-year period. Percentage values refer to the percent reduction in dengue cases compared with dengue incidence in the prevaccine era. Each vaccination strategy is indicated by the age of routine vaccination with its coverage levels and the target ages in the catch-up campaign. For example, R9 (70%) and 9–25 refer to a catch-up campaign of individuals from 9- to 25-year-olds followed by routine vaccination at 9-year-olds with a coverage level of 70%. For all catch-up campaigns considered, 50% vaccine coverage was assumed.

Our calculated values for the annual dengue infection incidence in the absence of vaccination (2.03%) and the annual symptomatic DF incidence (1.07%) were comparable to empirical estimates (Figures 1

and 2

**Figure 2.**
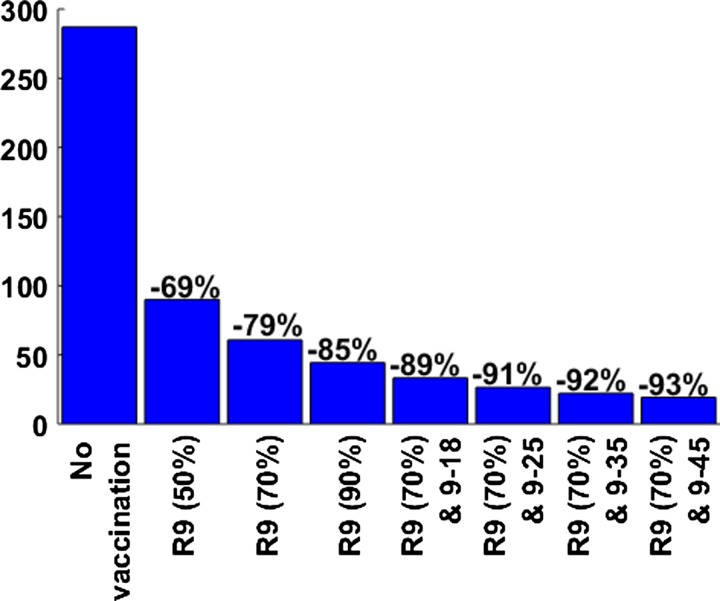
Expected yearly incidence of dengue hemorrhagic fever per million for the different vaccination strategies. Incidence was averaged over a 10-year period. Percentage values refer to the percent reduction in dengue cases compared with dengue incidence in the prevaccine era. Each vaccination strategy is indicated by the age of routine vaccination with its coverage levels and the target ages in the catch-up campaign. For example, R9 (70%) and 9–25 refers to a catch-up campaign of individuals from 9- to 25-year-olds followed by routine vaccination at 9-year-olds with a coverage level of 70%. For all catch-up campaigns considered, 50% vaccine coverage was assumed.

).^18^,^35^,^43^ The transmission rates (β_*k*_) were chosen to capture the age distribution among the cases of dengue in Brazil; the simulated age distributions of symptomatic cases of dengue in the prevaccine era are presented (Figure 3

**Figure 3.**
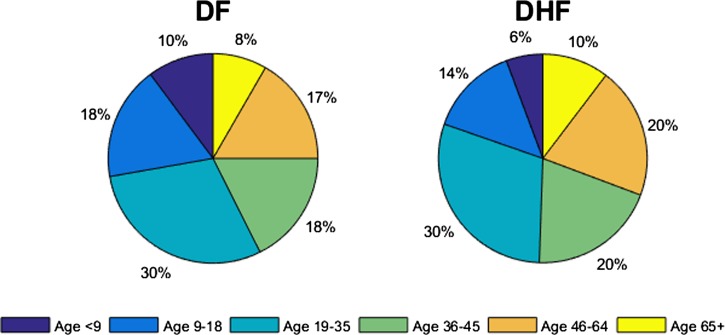
Age distributions of dengue fever (DF) and dengue hemorrhagic fever (DHF) cases in the prevaccine era. Age-specific incidence rates of DF and DHF cases in the prevaccine era are presented. An annual incidence of dengue fever and DHF cases are 1.07% and 0.029%, respectively.

). Before the dengue vaccine was introduced, the annual DHF incidence in Brazil was estimated to be 0.029%, resulting in a total cost of dengue at $906 million to the health-care system.

Our model shows, however, that the epidemiological burden associated with dengue would be significantly reduced by vaccination (Figures 1 and 2). Figure 1 presents the 10-year impact of each vaccination scenario in terms of the percent reduction of all symptomatic cases of dengue. The reduction in the incidence of dengue would range from 69% with a routine vaccination of 9-year-olds and 50% vaccine coverage to 93% with one year of a catch-up campaign targeting 9- to 45-year-olds with 50% vaccine coverage followed by a routine vaccination at 9-year-olds. This means that the number of dengue cases prevented over a 10-year period compared with the situation without vaccination would range from 14,800,000 with routine vaccination of 9-year-olds (50% vaccine coverage) to 19,979,900 with a catch-up campaign of 9- to 45-year-olds followed by routine vaccination of 9-year-olds (70% vaccine coverage level). Similarly, the number of DHF cases prevented over a 10-year period with a vaccination program is estimated to range from 394,024 with routine vaccination of 9-year-olds (50% vaccine coverage level) to 535,660 with a catch-up campaign for 9- to 45-year-olds followed by routine vaccination of 9-year-olds (70% vaccine coverage).

**Figure 4.**
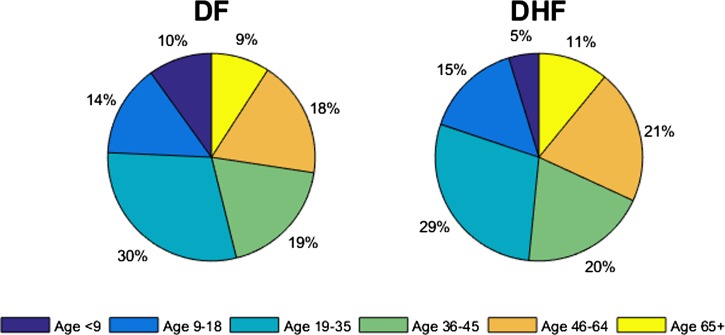
Age distributions of dengue fever (DF) and dengue hemorrhagic fever (DHF) cases after a 10-year period when routine vaccination of 9-year-olds are in practice. After 10 years of routine vaccination of 9-year-olds, the relative incidence of DF decreased among 9- to 18-year-olds by 4%, whereas it increased among individuals over 36–year-olds. The relative incidence of DHF increased by 1% in 9- to 18-year-olds and those over 36-year-olds.

In addition, we examined the age-specific impact of dengue vaccination with respect to the age distributions of DF and DHF cases in the pre- and postvaccine eras (Figure 3 and 4

). We found that after 10 years of routine vaccination of 9-year-olds, 14% of DF cases would occur among individuals aged 9–18, but in the prevaccine era, 18% of DF cases occurred in the same age group. However, the relative incidence of DHF increased by 1% in 9- to 18-year-olds and those over 36-year-olds.

**Figure 5.**
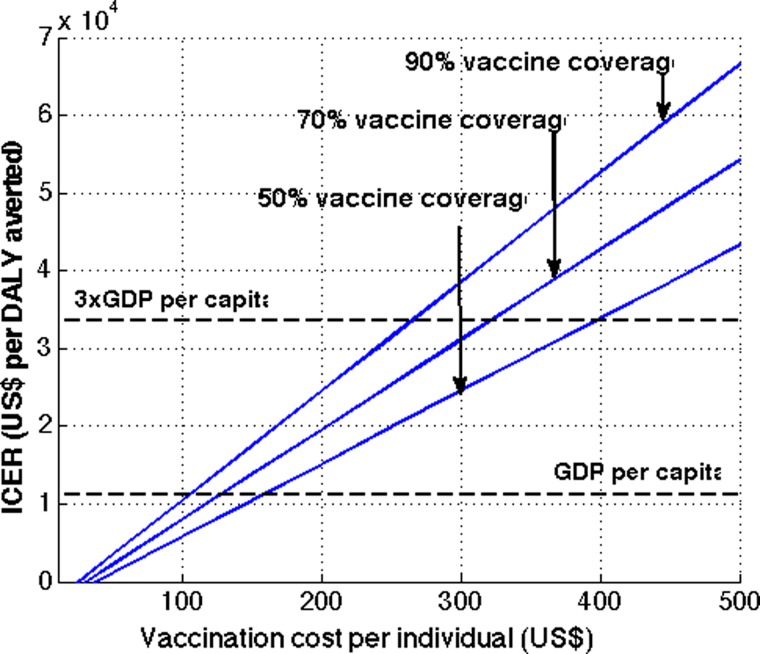
Cost-effectiveness of routine dengue vaccination with various coverage levels. The routine dengue vaccination of 9-year-olds was considered with 50%, 70%, and 90% vaccine coverage levels.

We evaluated the vaccine programs' cost-effectiveness with increasing cost per course of vaccination (Figure 5

and 6

**Figure 6.**
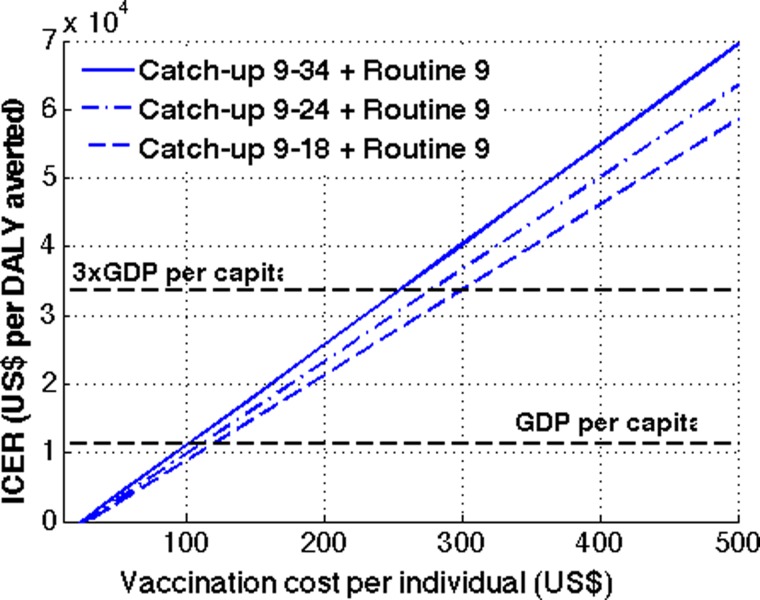
Cost-effectiveness of a catch-up campaign followed by routine dengue vaccination. One year of a catch-up campaign with various target ages (9–18, 9–24, and 9–34) followed by routine dengue vaccination of 9-year-olds was considered. For catch-up campaigns, 50% vaccine coverage was assumed, whereas 70% vaccine coverage was used for routine vaccination.

). In general, dengue vaccination in Brazil was more cost-effective with lower vaccine coverage levels. Specifically, with 50% vaccine coverage for the routine vaccination program, the cost per QALY gained from the health-care perspective is less than the $11,208 GDP per capita in Brazil when the cost of vaccination is $100 or lower, making the dengue vaccination program a very cost-effective intervention (Figure 5). Such a threshold cost of dengue vaccination per person, which allows the vaccination program to be very cost-effective, increases to $130 and to $160, when the vaccine coverage level increases to 70% and 90%, respectively. The threshold price per course of vaccination for which the cost of vaccination equaled three times the GDP is $262 for routine vaccination of 9-year-olds for 90% vaccine coverage. Therefore, a routine dengue vaccination of 9-year-olds would be cost-effective when the cost of vaccination per individual is < $262 for a vaccine coverage level of 90% or lower. For catch-up campaigns followed by routine vaccination, the cost-effectiveness decreases with wider target age groups (Figure 6). At a vaccination cost of $255 or lower, all catch-up campaigns considered were cost-effective.

## Discussion

The first dengue vaccine has now been approved for use in 13 countries. However, the impact of the new dengue vaccine might be hampered by the risk of immune-mediated enhancement of disease. Specifically, the trials of CYD-TDV revealed much lower efficacy in recipients who were seronegative at the time of vaccination than in those who were seropositive to dengue virus at the time of vaccination. As a result, lower vaccine efficacies were observed in younger age groups that have not lived long enough to experience a natural infection.

Even with the relatively low vaccine efficacy estimated in the recent dengue vaccine trials, our results show that age-targeted vaccination may still be cost-effective in Brazil. We show that routine vaccination of 70% of 9-year-olds would reduce the incidence of dengue infection by 79% and would be cost-effective across a range of vaccination costs. Achieving this level of vaccination coverage is expected to be feasible, given the high levels of adherence to childhood vaccination schedules in Brazil. Routine vaccination policies with higher vaccine coverage levels (90%) would result in higher cost-effectiveness ratios, but would be still considered cost-effective as long as the cost per course of vaccination is under $262. Similarly, for vaccination policies with a catch-up campaign, the cost-effectiveness of which was shown to be dependent on the targeted ages. If wider target ages were selected for a catch-up campaign, the cost-effectiveness of the vaccination program would be reduced, because the expansion of coverage would have the greatest health impact at the lowest coverage due to the decreasing marginal returns of herd immunity. Nevertheless, policies that combine routine coverage with onetime mass vaccination of vaccine-eligible age groups (i.e., ages 9–45) would have the highest impact of reducing the incidence of both DF and DHF by minimizing the lag time before population immunity is established.

Although our model incorporates some of key features of the newly developed dengue vaccine, such as vaccine-induced ADE and reduced efficacy in seronegative recipients, our analysis also has several limitations. In our model, serotype-specific efficacy parameters were not estimated. This is because country- and serotype-specific trial data are not currently publicly available; these data are necessary to estimate serotype-specific vaccine efficacy by fitting the observed serotype-specific attack rates.^8^,^42^ Second, outcomes measured in the dengue vaccine trials were based on clinically apparent disease, so it is currently unclear whether the vaccine protects against infection or just against disease.^56^ Our model assumes that both effects of the vaccine occur, that is, protection against infection as well as reduction in disease risk. Future expansions of our model could investigate the effects of various vaccine mechanisms on population protection and on the cost-effectiveness of a vaccination program. Furthermore, the development of agent-based model of dengue transmission and its use in the cost-effectiveness analysis would capture the spatial and temporal heterogeneity.

The incidence of dengue and its dispersion are rising due to climate changes, population growth, urbanization, and globalization. We evaluated the cost-effectiveness of various vaccination strategies in Brazil in light of the recent dengue vaccine approval. We show that carefully targeted vaccination would be cost-effective as a prevention strategy in Brazil and holds a potential to reduce the overall epidemiological and economic burden of dengue in the country.

## Supplementary Material

Supplemental Datas.

## Figures and Tables

**Table 1:** Model variables

Symbol	Variable
*S*_*k*_	Number of susceptible unvaccinated individuals in age group *k*
*I*_*k*_	Number of primarily infected unvaccinated individuals in age group *k*
*C*_*k*_	Number of unvaccinated individuals recovering from primary infections who are temporarily protected against clinical disease, in age group *k*
*R*_*k*_	Number of unvaccinated individuals susceptible to secondary infections in age group *k*
*Y*_*k*_	Number of unvaccinated individuals with secondary infections in age group *k*
*W*_*k*_	Number of unvaccinated individuals recovering from secondary infections in age group *k*
*P*_*k*_	Number of unvaccinated individuals recovering from secondary infections who are temporarily protected against clinical disease in age group *k*
*J*_*k*_	Number of unvaccinated individuals with tertiary infections in age group *k*
*Z*_*k*_	Number of unvaccinated individuals recovering from tertiary infections in age group *k*
*V*_*k*_	Number of partially susceptible vaccinated individuals in age group *k*
VI_*k*_	Number of primarily infected vaccinated individuals in age group *k*
VC_*k*_	Number of vaccinated individuals recovering from primary infections and temporarily protected against clinical disease in age group *k*
VR_*k*_	Number of vaccinated individuals susceptible to secondary infections in age group *k*
VY_*k*_	Number of vaccinated individuals with secondary infections in age group *k*
VW_*k*_	Number of vaccinated individuals recovering from secondary infections in age group *k*

**Table 2:** Epidemiological parameters

Symbol	Parameter	Value	References
*N*_*k*_	Relative size of age group *k*	*N*_1_ = 0.0723, *N*_2_ = 0.0628, *N*_3_ = 0.0157, *N*_4_ = 0.0900, *N*_5_ = 0.0713, *N*_6_ = 0.1082, *N*_7_ = 0.1722, *N*_8_ =0.1410, *N*_9_ = 0.1884, *N*_10_ = 0.0781	53
*b*_*k*_	Birth rate in Brazil in age group *k*	*b*_1_ = 3.9616 × 10^−5^	53
		*b*_*k*_ = 0 for *k* ≠ 1	
*p*_*k*_	Rate of aging out of age group *k* (*p*_*k*_ = 1/*a*_*k*_ where *a*_*k*_ is the age interval in age group *k*)	*p*_1_ = 0.0005, *p*_2_ = 0.0007, *p*_3_ = 0.0027, *p*_4_ = 0.0005, *p*_5_ = 0.0007, *p*_6_ = 0.0005, *p*_7_ = 0.0003, *p*_8_ = 0.0003, *p*_9_ = 0.0001, *p*_10_ = 0.0003	–
μ_*k*_	Death rate in age group *k*	μ_1_ = *b*/*N*_1_ − *p*_1_	–
		μ_*k*_ = *p*_*k* − 1_*N*_*k* − 1_/*N*_*k* − 1_*p*_*k*_ (*k* ≠ 1)		
β_*k*_	Transmission rate among age group *k*	β_1_ = 0.1256, β_2_ = 0.1209, β_3_ = 0.1302, β_4_ = 0.1488, β_5_ = 0.1953, β_6_ = 0.1767, β_7_ = β_8_ = 0.1860, β_9_ = β_10_ = 0.2325, β_11_ = β_12_ = β_13_ = β_14_ = 0.1860, β_15_ = 0.2418	Data fitting
σ_*n*_	Relative probability of being susceptible to *n*th infection	(5 − *n*)/4	18
ϕ_*k*_	Vaccination rate in age group *k*	ϕ_3_ = 0.00174 and ϕ_*k*_ = 0 for *k* ≠ 3 for Strategy A	Author's assumption
		ϕ_3_ = ϕ_4_ = 0.00174 and ϕ_*k*_ = 0 for *k* ≠ 3 or 4 for Strategy B	
*z*_*R*_	Wastage rate for routine vaccination program	10%	34,36
*z*_*C*_	Wastage rate for catch-up campaign	5%	34,36
ε	Vaccine efficacy against infection among the seronegative aged nine and over	0.616	12
δ	Vaccine efficacy against infection among the seropositive aged nine and over	0.792	12
*g*_*x*_	Proportion of dengue infections that are symptomatic in the epidemiological class *x*_*k*_	0.45 for *x*_*k*_ = *I*_*k*_	8
		0.8 for *x*_*k*_ = *Y*_*k*_ or VI_*k*_	
		0.14 for *x*_*k*_ = *J*_*k*_ or VY_*k*_	
*y*	Fraction of DF cases that sought medical care	0.5	37,38
γ	Rate of recovery from infection	0.146/day	30
γ_*C*_	Rate of loss of cross-immunity	0.0055/day	35,39
*h*_*x*_	Probability of developing DHF/DSS after symptomatic infection among the individuals in the epidemiological class *x*_*k*_	0.045 for *x*_*k*_ = *Y*_*k*_ or VI_*k*_	8
		0.25*h*_*Y*_ for *x*_*k*_ = *I*_*k*_, *J*_*k*_ or VY_*k*_	
χ	Risk of death from DHF/DSS	0.01	18,40

DF = dengue fever; DHF = dengue hemorrhagic fever; DSS = dengue shock syndrome; parameter values were used in the analysis unless indicated otherwise.

**Table 3:** Probabilities and costs of dengue infection

	Probability	Relative probability	Direct costs ($)	References
Dengue infection in the epidemiological class *x*_*k*_	1.00			
Asymptomatic	1 − *g*_*x*_			3
Symptomatic	*g*_*x*_	1.00		3
DF		1 − *q*_*x*_		18,28,29
Ambulatory		*Y* (1 − *q*_*x*_)	72	43
Severe (DHF)		*q*_*x*_		18,28,29
Hospitalized		(1 − χ) *q*_*x*_	267	43
Death		χ *q*_*x*_	NA	43

DF = dengue fever; DHF = dengue hemorrhagic fever; NA = non applicable; all values are reported in 2017 U.S. dollars.

**Table 4:** Cost-effectiveness parameters

Symbol	Parameter	Value	References
*r*	Social discount rate for QALYs calculations	0.03	17,44
*D*_Death_	Disability weight for death	1	17,44
*D*_DF_	Disability weight for DF	0.197	18,45
*D*_DHF_	Disability weight for DHF/DSS	0.545	18,45
*L*_DF_	Time lost due to DF (years)	0.019	18,46–48
*L*_DHF_	Time lost due to DHF/DSS (years)	0.0325	18,47
*L*_Death, *k*_	Years of life lost due to death for age group *k*	67.5 for *k* = 1, 63 for *k* = 2, 61 for *k* = 3, 57.5 − 5 (*k* − 4) for *k* = 4, …, 15	
*a*_*k*_	Average age of dengue exposure in age class *k*	2.5 for *k* = 1, 7 for *k* = 2, 9 for *k* = 3, 5 (*k* − 4) + 12.5 for *k* = 4, …, 15	

DF = dengue fever; DHF = dengue hemorrhagic fever; DSS = dengue shock syndrome; QALY = quality-adjusted life-years.
