# Supplementary material for: Cost-Effectiveness of Dengue Vaccination Programs in Brazil
Source: Am J Trop Med Hyg. 2017 May 3;96(5):1227–34. doi: 10.4269/ajtmh.16-0810 (PMC5417221; doi:10.4269/ajtmh.16-0810)
Supplement: Supplementary file 1 [file SD9.pdf]

SUPPLEMENTAL APPENDIX

MATHEMATICAL MODEL OF DENGUE TRANSMISSION AND VACCINATION

In our model, the population comprises 15 distinct age classes, which represent individuals aged 0–4, 5–8, 9, 10–14, 15–19, 20–25, . . ., 60–64, and 65 and over. The relative size of the age groups represents the current age structure in Brazil. Within each age class, we incorporate 15 epidemiological classes. Detailed descriptions of the epidemiological classes can be found in Table 1. Using these notations, the age-structured model of dengue transmission and vaccination is given by:

$$\frac{dS_k}{dt} = b_k + {}^{k}p_{k-1}S_{k-1} - (\varphi_k + \sigma_1\lambda_k + \mu_k + p_k)S_k,$$

$$\frac{dI_k}{dt} = p_{k-1}I_{k-1} + \sigma_1\lambda_k S_k - [\gamma + (1-g)\varphi_k + \mu_k + p_k]I_k,$$

$$\frac{dC_k}{dt} = p_{k-1}C_{k-1} + \gamma I_k - (\gamma_C + \varphi_k + \mu_k + p_k)C_k,$$

$$\frac{dR_k}{dt} = p_{k-1}R_{k-1} + \gamma_C C_k - (\sigma_2\lambda_k + \varphi_k + \mu_k + p_k)R_k,$$

$$\frac{dY_k}{dt} = p_{k-1}Y_{k-1} + \sigma_2\lambda_k R_k - [\gamma + (1-g)\varphi_k + \mu_k + p_k]Y_k,$$

$$\frac{dW_k}{dt} = p_{k-1}W_{k-1} + \gamma Y_k - (\gamma_C + \mathrm{f}_k + \mu_k + p_k)W_k,$$

$$\frac{dP_k}{dt} = p_{k-1}P_{k-1} + \gamma_C W_k - (\sigma_3\lambda_k + \varphi_k + \mu_k + p_k)P_k,$$

$$\frac{dJ_k}{dt} = p_{k-1}J_{k-1} + \sigma_3\lambda_k P_k - (\gamma + \varphi_k + \mu_k + p_k)J_k,$$

$$\frac{dZ_k}{dt} = p_{k-1}Z_{k-1} + \gamma J_k - (\varphi_k + \mu_k + p_k)Z_k,$$

$$\frac{d\mathrm{VS}_k}{dt} = p_{k-1}\mathrm{VS}_{k-1} + \varphi_k S_k - \sigma_1\lambda_k(1-\varepsilon)\mathrm{VS}_k - (\mu_k + p_k)\mathrm{VS}_k,$$

$$\frac{d\mathrm{VI}_k}{dt} = p_{k-1}\mathrm{VI}_{k-1} + (1-g)\varphi_k I_k + \sigma_1\lambda_k(1-\varepsilon)\mathrm{VS}_k - (\gamma + \mu_k + p_k)\mathrm{VI}_k,$$

$$\frac{d\mathrm{VC}_k}{dt} = p_{k-1}\mathrm{VC}_{k-1} + \varphi_k C_k + \gamma \mathrm{VI}_k - (\gamma_C + \mu_k + p_k)\mathrm{VC}_k,$$

$$\frac{d\mathrm{VR}_k}{dt} = p_{k-1}\mathrm{VR}_{k-1} + \varphi_k R_k + \gamma_C \mathrm{VC}_k - \sigma_2\lambda_k(1-\delta)\mathrm{VR}_k - (\mu_k + p_k)\mathrm{VR}_k,$$

$$\frac{d\mathrm{VY}_k}{dt} = p_{k-1}\mathrm{VY}_{k-1} + (1-g)\varphi_k Y_k + \sigma_2\lambda_k(1-\delta)\mathrm{VR}_k - (\gamma + \mu_k + p_k)\mathrm{VY}_k,$$

$$\frac{d\mathrm{VW}_k}{dt} = p_{k-1}\mathrm{VW}_{k-1} + \varphi_k(W_k + P_k + J_k + Z_k) + \gamma \mathrm{VY}_k - (\mu_k + p_k)\mathrm{VW}_k$$

where

$$\lambda_k = \frac{\beta_k \sum_{k=1}^{6}(I_k + Y_k + J_k + \mathrm{VI}_k + \mathrm{VY}_k)}{N}$$

and $\sigma_n = (5 - n)/4$.

In our model, unvaccinated individuals who recover from third infections (Z_k) or vaccinated individuals who recover from secondary infections (VW_k) are assumed to be immune to all strains. The subscript k in the equations refers to the age groups k. The rates of birth and death are denoted by b_k and μ_k, respectively ($b_k = 0$ if $k \neq 1$). The rate p_k is the rate at which individuals leave an age group k and enter age group $k + 1$ through aging with $p_0 = p_{15} = 0$.

CALCULATION OF QUALITY-ADJUSTED LIFE YEARS AND COSTS ASSOCIATED WITH DENGUE

We measured the effectiveness of each strategy in quality-adjusted life years (QALYs) to account for both time and quality of life. Specifically, we calculated the time-discounted QALYs lost to (DF), dengue hemorrhagic fever dengue fever (DHF)/dengue shock syndrome , and dengue-related deaths. A disability weight of one was used for premature death. The rate of new DF cases, DHF cases, and dengue-related deaths (Death_k) in age group k was calculated as following:

$$\frac{d\mathrm{DF}_k(t)}{dt} = g_I(1-q_I)\lambda_k S_k + g_Y(1-q_Y)\lambda_k R_k + g_J(1-q_J)\lambda_k P_k + (1-\varepsilon)g_{\mathrm{VI}}(1-q_{\mathrm{VI}})\lambda_k \mathrm{VS}_k + (1-\delta)g_{\mathrm{VY}}(1-q_{\mathrm{VY}})\lambda_k \mathrm{VR}_k,$$

$$\frac{d\mathrm{DHF}_k(t)}{dt} = g_I q_I \lambda_k S_k + g_Y q_Y \lambda_k R_k + g_J q_J \lambda_k P_k + (1-\varepsilon)g_{\mathrm{VI}} q_{\mathrm{VI}} \lambda_k \mathrm{VS}_k + (1-\delta)g_{\mathrm{VY}} q_{\mathrm{VY}} \lambda_k \mathrm{VR}_k,$$

$$\frac{d\mathrm{Death}_k(t)}{dt} = \mathrm{X}\frac{d\mathrm{DHF}_k(t)}{dt}$$

Using the equations above as well as the following equation, we calculated the number of dengue episodes and QALYs lost in each case[1–3]:

$$\int_0^{T_f} e^{-rt}\sum_{k=1}^{15}\left[\Delta Q_{\mathrm{DF}}\left(\frac{d\mathrm{DF}_k(t)}{dt}\right) + \Delta Q_{\mathrm{DHF}}\left(\frac{d\mathrm{DHF}_k(t)}{dt} - \frac{d\mathrm{Death}_k(t)}{dt}\right) + \frac{Q_{\mathrm{No\ Disease}}}{r}\left(1 - e^{-rL(k,\mathrm{No\ Disease})}\right)\left(\frac{d\mathrm{Death}_k(t)}{dt}\right)\right]dt.$$

Here, $Q_{\mathrm{No\ Disease}}$ is the quality of life in the absence of dengue infection (assumed to be one), $L(k, \mathrm{No\ Disease})$ is the residual expected lifespan of an individual in the age group k in the absence of dengue infection, r is the social discount rate of 3%, and Q_{DF} and Q_{DHF} are the quality of life lost per episode of dengue fever and DHF (Table 2). To calculate the health effects, the quality-adjusted life expectancy (QALE) was first calculated in the case of a lethal dengue infection as

Discounted QALE, at age a with disease status,

$$D = Q_D\frac{1 - e^{-rL(a,D)}}{r},$$

where Q_D is the quality of life associated with a disease state (D) and L is the residual life expectancy for an individual considering the life expectancy in the Philippines is 70 years.[3,4] Therefore, the discounted QALY loss at

age a, associated with dengue-related deaths, can be calculated as

$$\Delta Q_D \frac{1-e^{-rL(a,D)}}{r} = Q_{\text{No Disease}} \frac{1-e^{-rL(a,\text{No Disease})}}{r} - Q_{\text{Death}} \frac{1-e^{-rL(a,\text{Death})}}{r} = Q_{\text{No Disease}} \frac{1-e^{-rL(a,\text{No Disease})}}{r}.$$

For the associated nonlethal infections, the QALY loss for DF and DHF is Q_{DF} and Q_{DHF}, respectively.

In addition, the total costs accrued due to medical treatment, vaccination, and lost productivity is estimated by the following:

$$\text{Costs} = \text{costs of vaccination} + \text{costs associated with dengue infection (DH and DHF)}$$

$$= \int_0^{T_f} \left\{ \begin{array}{l} \sum_{k=1}^{6} C_{V,k}\varphi_k (S_k + (1-g)I_k + C_k + R_k + (1-g)Y_k + W_k + J_k + P_k + Z_k) \\ + \sum_{k=1}^{11} \left(C_{\text{DF},k} y \frac{\text{dDF}_k(t)}{\text{d}t} + C_{\text{DHF},k} \frac{\text{dDHF}_k(t)}{\text{d}t} \right) \end{array} \right\} e^{-rt} \text{d}t$$

For the cost-effectiveness analysis from the health-care perspective, only direct costs were considered in the above equation.

SUPPLEMENTAL REFERENCES

1. Carrasco LR, Lee LK, Lee VJ, Ooi EE, Shepard DS, Thein TL, Gan V, Cook AR, Lye D, Ching Ng L, Leo YS, 2011. Economic impact of dengue illness and the cost-effectiveness of future vaccination programs in Singapore. *PLoS Negl Trop Dis 5:* e1426.
2. Murray CJ, 1994. Quantifying the burden of disease: the technical basis for disability-adjusted life years. *Bull World Health Organ 72:* 429–445.
3. Shim E, Galvani AP, 2009. Impact of transmission dynamics on the cost-effectiveness of rotavirus vaccination. *Vaccine 27:* 4025–4030.
4. Atkins KE, Shim E, Carroll S, Quilici S, Galvani AP, 2012. The cost-effectiveness of pentavalent rotavirus vaccination in England and Wales. *Vaccine 30:* 6766–6776.

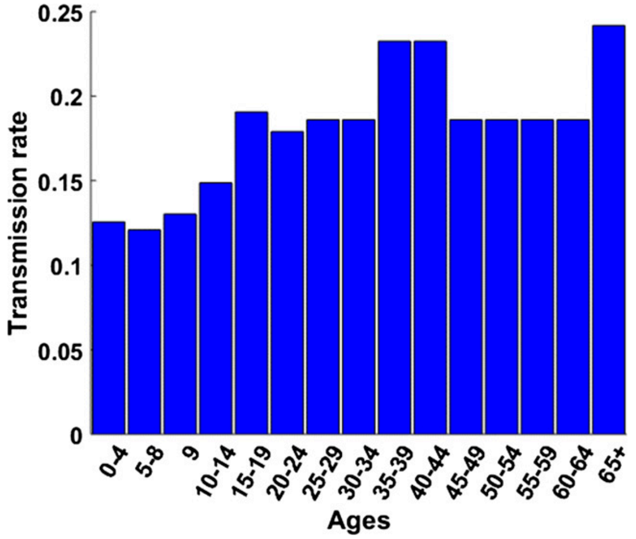

SUPPLEMENTAL FIGURE 1. Transmission rate among age groups.
